# An unusual form of kidney injury without glomerulonephritis in microscopic polyangiitis: a case report

**DOI:** 10.1186/s12882-023-03134-0

**Published:** 2023-03-31

**Authors:** Cihan Uysal, Tugba Yilmaz, Hafsa Kocyigit, Hulya Akgun, Murat Hayri Sipahioglu

**Affiliations:** 1 Department of Nephrology, Erciyes University Medical School, Dede Efendi Sreet, Köşk District, 38030 Melikgazi, Kayseri Turkey; 2 Department of Internal Medicine, Kayseri State Hospital, Kayseri, Turkey; 3 Department of Pathology, Erciyes University Medical School, Kayseri, Turkey

**Keywords:** Tubulointerstitial nephritis, Microscopic polyangiitis, Acute kidney injury, Dialysis, Case report

## Abstract

**Background:**

Microscopic polyangiitis (MPA), a kind of antineutrophil cytoplasmic autoantibody associated vasculitis (AAV), predominantly affects small-sized vessels. MPA is a significant cause of the pulmonary-renal syndrome. Pauci-immune necrotizing and crescentic glomerulonephritis is the typical renal histological feature of AAV. Tubulointerstitial lesions may occur and mostly form with inflammatory cell infiltration in the interstitium. However, a few cases reported only tubulointerstitial involvement without glomerular lesions in patients with MPA.

**Case presentation:**

We present an MPA case, a 70-year-old male patient diagnosed with acute kidney injury accompanying the dialysis requirement. Only acute tubulointerstitial nephritis was revealed in kidney biopsy without evidence of glomerular injury. Also, interstitial pulmonary fibrosis was determined on computerized tomography, and myeloperoxidase antineutrophil cytoplasmic autoantibody was positive. Consequently, we have considered the main diagnosis as MPA. We did not prefer a standard tubulointerstitial nephritis treatment regimen due to the presence of life-threatening systemic vasculitis. Treatment was established like crescentic glomerulonephritis. Induction therapy consisted of pulse steroid, cyclophosphamide, and plasmapheresis. Unfortunately, severe SARS-CoV-2 infection caused death during induction therapy in this case.

**Conclusions:**

The lack of glomerular injury and solely interstitial inflammation is atypical regarding AAV involvement in the kidney. This diversity might be initially considered as only a simple histological elaboration. However, it is a significant entity for guiding the treatment of AAV.

## Background

Antineutrophil cytoplasmic autoantibody (ANCA)-associated vasculitis (AAV) is a group of disorders that affect small-sized vessels predominantly and have similar histopathological features in kidney involvement [1]. Microscopic polyangiitis (MPA), a kind of AAV, is a significant cause of pulmonary-renal syndrome [2]. Although MPA has been reported at all ages, intensive and severe clinics generally occur in older adults [3]. Clinical manifestations are various in MPA; most of the patients present with constitutional symptoms including; fatigue, fever, arthralgia, or weight loss at diagnosis or months prior to diagnosis [3]. The hazard of respiratory failure due to severe pulmonary hemorrhage and permanent kidney dysfunction due to rapidly progressive glomerulonephritis (RPGN), therefore prompt identification of the disease, is critical. Furthermore, these consequences can be prevented by proper treatment. The treatment of MPA includes vigorous immunosuppression strategies. ANCA test has diagnostic significance on AAV. The immunofluorescence assay (IFA) usually has a perinuclear staining pattern (P-ANCA), and enzyme-linked immunoassays (ELISA) detect antibodies to myeloperoxidase (MPO-ANCA) in MPA.

Kidney involvement is noted in approximately 80-100% of patients with MPA cases, and the clinic spectrum ranges from microscopic hematuria to severe kidney injury with dialysis requirement [4]. Kidney involvement of MPA presents with evident glomerulonephritis (GN) in almost all cases. Pauci-immune necrotizing and crescentic GN is the typical histological feature in AAV [5]. Nevertheless, renal involvement of systemic vasculitis occasionally may be in the form of tubulointerstitial nephritis (TIN).

Acute interstitial nephritis is a kidney pathology that typically leads to a decrease in kidney function and is characterized by an inflammatory cell infiltration in the interstitium. It is frequently induced by drug therapy. Also, caused by autoimmune disorders, systemic disease, several infections, and tubulointerstitial nephritis with uveitis (TINU) syndrome [6]. However, a few cases were reported, a purely tubulointerstitial injury without glomerular lesions in patients with MPA [7–9]. Herein, we describe life-threatening pulmonary-renal syndrome accompanied by an unexpected renal component. We present an unusual form of kidney injury by MPA, which resulted in dialysis requirements.

## Case presentation

A 70-year-old male patient was admitted to the emergency department with confusion and vomiting. He had no antecedent chronic disease in his past medical history. He had not been taking any prescribed medications. His complaints had started 2 weeks ago. First, a neurologist had examined him for headaches and insomnia. Any remarkable disorder had not been detected in the first physical examination. Only increased blood pressure (155/95 mmHg) had been observed. Any significant lesion had not been determined in the central nervous system imaging. Also, the major abnormality had not been detected in laboratory assessment, and the serum creatinine level had been 0,8 mg/dl. Only a salt-free diet had been recommended to him for new-onset hypertension. However, the clinial status of the patient had deteriorated over time at home. Afterward, he had been transported to the hospital by ambulance due to impaired consciousness.

On physical examination, he was confused, tachypneic (24/minute), tachycardic (106/minute), and hypertensive (170/95 mmHg). The mouth was dry; a respiratory sound decreased on the lower right side, the abdominal examination was usual, and palpable purpura with pitting edema was observed on the lower extremities.

The second laboratory results were as follows; BUN:154 mg/dl, creatinine: 19,2 mg/dl, sodium: 138 mEq/L, potassium: 6,2 mEq/L, calcium: 9,5 mg/dl, phosphorus: 9,9 mg/dl, uric acid: 12 mg/dl, glucose: 82 mg/dl, protein: 5.6 g/dl, albumin: 2,8 g/dl, ALT: 25 IU/L, AST:13 IU/L, LDH: 201 IU/L, bilirubin: 0,4 mg/dl, CRP: 164 mg/L, leukocyte:14.2 10^3^/μl, hemoglobin: 8.2 g/dl, platelet: 390 10^3^/μl. The urine output was less than 100 ml. Emergency dialysis (two hours) was performed promptly due to hyperkalemia and uremic symptoms. Afterward, treatment and etiological investigation were maintained in the nephrology service.

The urine sediment analysis detected isomorphic erythrocytes, pyuria, granular cast, and leukocyte cast. The urinary protein-to-creatinine ratio was 0.8 mg/g. Any pathogen was not isolated in urine cultures. On ultrasonographic examination, kidney size and echogenicity were normal. The ophthalmologic examination has not shown signs of hypertensive retinopathy and uveitis.

**Fig. 1:**
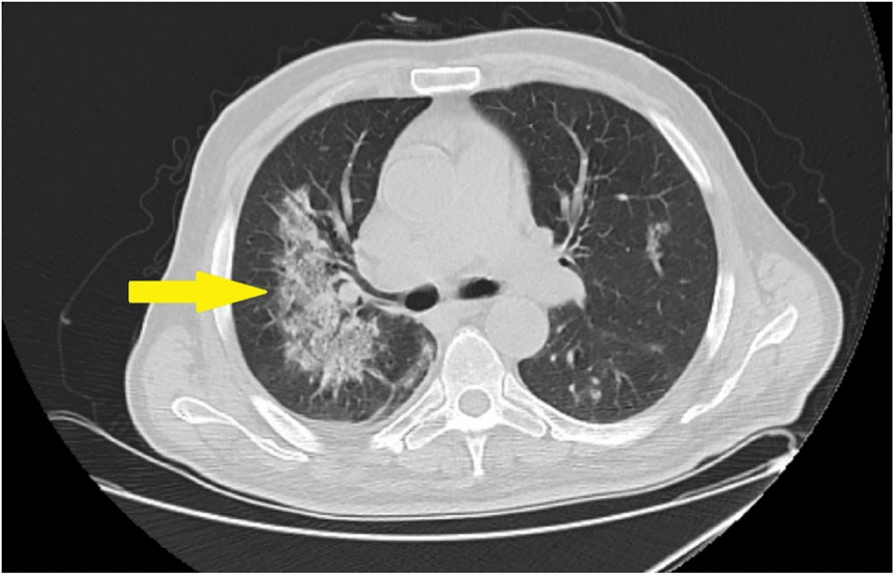
Thoracic Computerized Tomography Image. Yellow narrow shows interstitial fibrosis area due to vasculitis involvement

His clinical condition improved after the second dialysis session. Blood pressure was controlled, and the confusion resolved. Moreover, he restarted oral feeding. However, urine output did not increase and oliguria sustained. Afterward, hemoptysis onset on the third day of hospitalization. The chest imaging was performed promptly with thoracic computed tomography (CT). CT images revealed minimal pleural effusion and bilateral interstitial fibrotic areas; the image is shown in Fig. 1. These findings were reported in accordance with pulmonary involvement of vasculitis attack. Bronchoscopy was planned, however, the patient refused the intervention of the lung.

The immunoassay laboratory resulted in as follows; anti-glomerular basal membrane (GBM) antibody was negative, anti-nuclear antibodies (ANA) was positive with 1 /100 titer (granular pattern), anti-double-stranded DNA antibody was negative, anti-SSA / anti-SSB were negative, C3 / C4 levels were in the normal range, cytoplasmic ANCA (C-ANCA) was negative, P-ANCA was positive (formaline resistance). The ELISA test confirmed the positivity of perinuclear staining, with high titers MPO-ANCA (> 200 RU/ml). Lastly, serum protein electrophoresis did not show monoclonal gammopathy, and a serum-free light chain ratio (K/λ) was in a normal range.

**Fig. 2:**
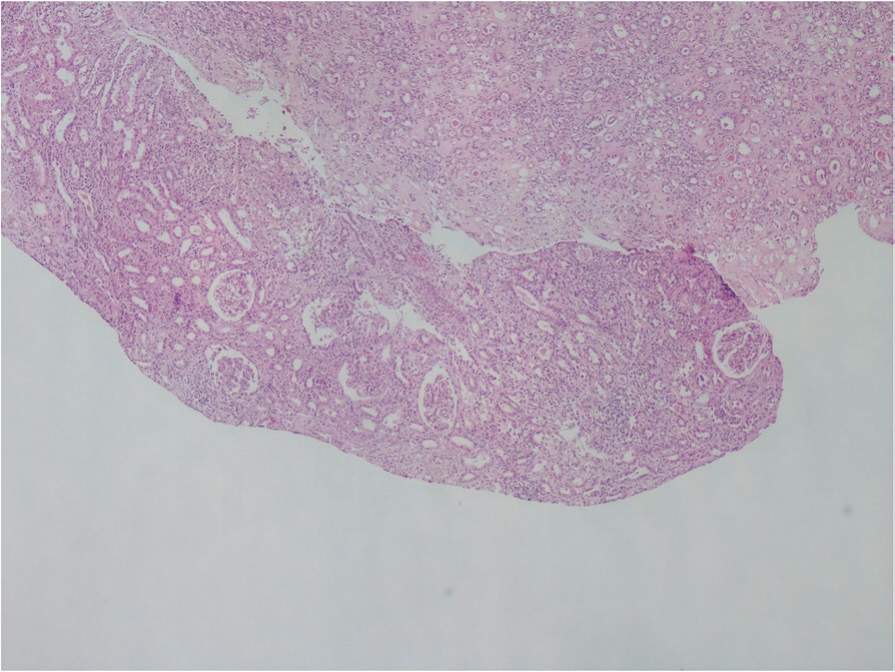
Kidney Biopsy Findings. Periodic-acid-Schiff staining section shows interstitial area is infiltrated by inflammatory cells and intact glomerulus

**Fig. 3:**
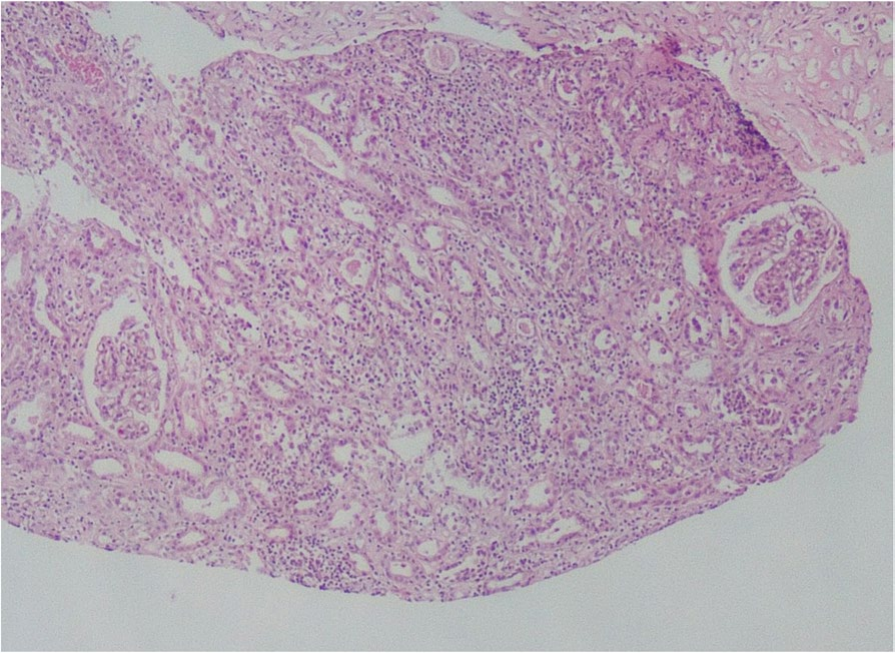
Kidney Biopsy Findings. Periodic-acid-Schiff staining section shows interstitial area is infiltrated by inflammatory cells, cast materials in tubular lumens, and no crescent formation on glomerulus

A kidney biopsy was performed after several dialysis sessions. The kidney biopsy was reported as acute tubulointerstitial nephritis, and the biopsy sections are shown in Figs. 2 and 3. The kidney biopsy findings were reported as follows. *The five glomeruli have been detected in light microscopy. The two glomeruli have been globally sclerotic, and the others appeared intact. The damaged tubular epithelial cells and tubular atrophy have been observed. Also, erythrocytes have been observed in tubular lumens. Hyaline arteriosclerosis has been detected in the vessel wall. Interstitial areas have been infiltrated by inflammatory cells, including neutrophils, eosinophils, lymphocytes, and plasma cells. The glomerular staining has not been shown in the immunofluorescence examination. Only tubular cast material has been abundantly stained with IgA, IgM, Kappa, and Lambda. Congo Red staining has resulted in negative.*

Consequently, we have decided to the MPA as a diagnosis. We preferred to a treatment similar to crescentic glomerulonephritis due to the life-threatening vasculitis. The induction regimen consisted of a combination. Pulse steroid therapy (500 mg methylprednisolone for 3 days) was administered and continued with an oral dose of 1 mg/kg/day. The parenteral cyclophosphamide (15 mg/kg for every 3 weeks) therapy was administered. We planned the duration of the cyclophosphamide regimen as 6 months. Seven plasmapheresis sessions were performed with Fresh Frozen Plasma (FFP). The patient was 72 kg, so approximately four liters of FFP were used for each session. The plasmapheresis was performed on an every-other-day basis. Clinical and radiological improvement was observed in the lung and skin lesions after induction therapy, although the dialysis requirement has remained.

Dialysis therapy continued three times a week after being discharged home. A total of 3 cycles of cyclophosphamide were administered. Despite the increased urine output, approximately 1 liter daily, estimated glomerular filtration rate (eGFR) was still below 10 ml/min/1.73 m2. Afterward, the patient was infected with SARS-CoV-2. He was hospitalized in the intensive care unit due to severe disease. Finally, acute respiratory failure led him to death. The exact duration between the diagnosis of AKI and the patient’s death was 92 days.

## Discussion and conclusions

We have presented a case of pulmonary-renal syndrome constituted by MPA. The kidney involvement has consisted of only tubulointerstitial nephritis (TIN) in this reported case. This state is unexpected and rare for AAV. Also, severe kidney injury accompanied by dialysis requirement has occurred through this injury mechanism. This is a special condition for severe pulmonary-renal syndrome and has not been reported in the literature before. Renal manifestations have been reported in almost 100% of patients with MPA. Severe kidney injury due to RPGN, is the essential clinical feature of MPA. Despite significant changes demonstrated in the glomerular apparatus, damage to tubular and interstitial can be part of the kidney involvement [10]. The lack of glomerular injury and solely interstitial inflammation is atypical regarding AAV involvement. However, several cases have been reported [7–9, 11, 12].

In this case, we preferred conventional induction regimen of AAV due to life-threatening disease. Our approach to initial therapy depends upon the severity of the disease and the organ’s involvement. Active renal and pulmonary injuries were detected. Observational studies revealed that cyclophosphamide plus glucocorticoids as induction therapy was associated with a higher amelioration in survival and a lower frequency of relapse [13]. Also, Kidney Disease Improving Global Outcomes (KDIGO) guideline favors a cyclophosphamide-based regimen as initial therapy in serious kidney disease [14]. The patient was informed about the side effects of immunosuppression, the severity of the disease, and the risks of Coronavirus Disease 2019 (COVID-19) before the treatment. Nonetheless, this mortal consequence dejected us. In our opinion, the senility of the patient and the kidney disease contributed to poor prognosis. Lastly, cyclophosphamide has been associated with worse outcomes in COVID-19 [15].

Acute interstitial nephritis is characterized by an inflammatory infiltrate in the kidney interstitium. It is most often induced by drugs or systemic diseases such as Sjögren’s syndrome, sarcoidosis, and systemic lupus erythematosus. The differential diagnosis of interstitial nephritis in AAV was evaluated in this case. The autoimmune diseases were excluded via the lack of autoantibodies. The infectous factors were excluded via microbiological examination. Uvetitis was not detected. We did not consider sarcoidosis due to the lack of tissue granulomas and hypercalcemia. Our patient took only one paracetamol tablet 1 day before the emergency admission. This duration (1 day) is too short for the predicted onset of a drug-induced TIN and dialysis requirement. Paracetamol-associated TIN was reported in the literature [16]. However, severe AKI has been detected only 1 day after medication. Also, constitutional symptoms have already occurred before taking paracetamol. Therefore, we did not consider drug- related TIN.

The diagnosis of AAV is based upon the combination of characteristic clinical findings, pathological evidence, laboratory tests, and imaging studies. In the presented case, MPA diagnosis was established with of positive MPO-ANCA test, acute kidney injury, radiological lung lesions, and skin lesions. The conventional treatment of acute TIN has not been preferred for the patient. Due to life-threatening lung and kidney involvement, we administered AAV induction therapy [14].

The lack of histological evidence of vasculitis may conflict with the diagnosis. However, several laboratory and radiological evidence were essential in diagnosing AAV. Especially ANCA assays are a very reliable biomarker for AAV diagnosis. The relationship between ANCA with vasculitides was well established [4]. Approximately 90% of patients with MPA have ANCA positivity in widely used types of ANCA assessments. P-ANCA pattern results from a staining pattern around the nucleus on IFA. Most MPA patients have MPO-ANCA, but a small part has PR3-ANCA on ELISA [17]. Both MPO-ANCA and P-ANCA positivity has high specificity for MPA (P-ANCA 81-96% and MPO-ANCA 96-99%) [18]. ELISA should be performed to determine the specific antibody and subsequently positive IFA detection. In this case, formalin-fixed staining and high titer of MPO-ANCA on ELISA have been confirmed to the positivity of P-ANCA.

Pulmonary involvement is noted in approximately 25-55% of patients with MPA. Clinical manifestations include; alveolar hemorrhage, infiltrates, pulmonary edema, pleuritis, and interstitial fibrosis. Common presenting symptoms are dyspnea, cough, hemoptysis, and chest pain. The most common radiological finding is ground-glass attenuation, which corresponds to alveolar hemorrhage, chronic interstitial inflammation of the alveolar septa, and capillaritis [19]. CT evaluation revealed interstitial pneumonitis and septal thickening in this presented case.

MPA often has cutaneous signs scattered over the lower extremities. The skin lesions are mainly palpable purpura or histologically leukocytoclastic vasculitis induced by necrotizing capillaries [20]. The purpuric lesions were observed on the legs initially; these rashes disappeared after steroid therapy in this case.

We had planned to repeat the kidney biopsy in this case due to the remaining dialysis requirement. However, we could not perform because patient survival was too short. The low quantity of glomeruli in biopsy might have obscured glomerular injury. Also, the lack of electron microscopic examination is another deficiency of our approach. Another limitation of this case is the lack of IgG4 assessment. Indeed, the MPO positivity with high titers persuaded us to diagnosis.

In literature screening, we encountered a few cases that resemble this patient. Wen et al. have reported pulmonary-renal involvement of MPA; kidney biopsy showed all intact 25 glomeruli and infiltration of inflammatory cells in the tubular epithelium. The initial therapy has failed and consisted of only low-dose glucocorticoids. The clinical remission has attained subsequently after the administration of pulse steroid plus cyclophosphamide therapy [8]. Hassani et al. reported an MPA case accompanied by acute TIN without another systemic findings [11]. In another case with MPA, the first kidney biopsy has shown TIN without glomerular injury. After 6 years, the active glomerular injury has not shown again in the second biopsy. The only evidence of chronicle alterations have been detected, such as glomerulosclerosis, mild interstitial fibrosis, and tubular atrophy in the latter specimen [12]. In another case of drug-associated MPA, the patient had a positive MPO-ANCA test and a positive drug-induced lymphocyte stimulation test for cimetidine. Kidney biopsy has shown alterations consistent with acute TIN and intact 11 glomeruli. The kidney biopsy has been repeated after drug discontinuation, and only persistent TIN has been observed again [9]. Furthermore, isolated interstitial nephritis has been reported in granulomatosis polyangiitis (GPA) [21]. These aforementioned cases are summarized in Table 1.

**Table 1:** Reported cases of acute TIN associated with AAV

Authors	Age (years)	Sex	ANCA	Pulmonary Involvement	Dialysis Performed
Wen et al.	65	F	MPO	YES	NO
Plafkin et al.	45	F	MPO	NO	YES
Morimoto et al.	70	F	MPO	NO	NO
Fannin et al.	62	M	PR3	YES	NO
Hassani et al.	75	M	MPO	NO	NO
Kasahara et al.	74	F	MPO	NO	NO
This case	70	M	MPO	YES	YES

*Abbreviations*: *F* female, *M* male

Several observations and presumptions have been noted about TIN accompanying crescentic glomerulonephritis. For example, the rupture of Bowman’s capsule subsequently causes inflammation involves in the interstitial area. Also, the interstitial region-specific antigen’s avid affinity to autoantibodies has been proposed as a possible mechanism for originating TIN in MPA without glomerular injury [22]. The spillover of peritubular capillaritis and interstitial granuloma formation are other suppositions [23].

The pathogenesis of this atypical process in MPA is not elucidated currently, despite the increasing number of reported cases. This diversity may be considered as only a simple histological elaboration. However, it is a significant entity for guiding the treatment. Furthermore, this case emphasizes the presence of vasculitides in the etiology of TIN. We presented an unusual kidney involvement of MPA, so physicians should be alert regarding atypical vasculitis presentations.

## Data Availability

Further clinical data and images of this case are available from the corresponding author upon reasonable request.

## Abbreviations

ANCA: Antineutrophil cytoplasmic autoantibody
AAV: ANCA-associated vasculitis
ANA: Antinuclear antibodies
antiSSA: anti–Sjögren’s-syndrome-related antigen A autoantibodies
antiSSB: anti–Sjögren’s-syndrome-related antigen autoantibodies
ALT: Alanine aminotransferase
AST: Aspartate aminotransferase
BUN: Blood urea nitrogen
C-ANCA: Cytoplasmic staining ANCA
CRP: C-reactive protein
C3: Complement component 3
C4: Complement component 4
COVID-19: Coronavirus Disease 2019
CT: Computed tomography
DNA: Deoxyribonucleic acid
eGFR: Estimated glomerular filtration rate
ELISA: Enzyme-linked immunoassays
F: Female
FFP: Fresh frozen plasma
GBM: Glomerular basal membrane
GN: Glomerulonephritis
GPA: Granulomatosis polyangiitis
IFA: Immunofluorescence assay
IgA: Immunoglobulin A
IgM: Immunoglobulin M
IU: International unit
KDIGO: Kidney Disease Improving Global Outcomes
*κ/λ*: kappa to lambda ratio
LDH: Lactate dehydrogenase
M: Male
MPA: Microscopic polyangiitis
MPO: Myeloperoxidase
P-ANCA: Perinuclear staining ANCA
PR-3: Proteinase 3
RPGN: Rapidly progressive glomerulonephritis
RU: Relative units
SARS-CoV-2: Severe acute respiratory syndrome coronavirus 2
TIN: Tubulointerstitial nephritis
